# Effects of troxerutin on anxiety- and depressive-like behaviors induced by chronic mild stress in adult male rats

**DOI:** 10.22038/IJBMS.2018.26915.6582

**Published:** 2018-08

**Authors:** Maryam Azarfarin, Fereshteh Farajdokht, Shirin Babri, Farzad Salehpour, Milad Taghizadeh, Gisou Mohaddes

**Affiliations:** 1 Drug Applied Research Center, Tabriz University of Medical Sciences, Tabriz, Iran; 2 Higher Education Institute of Rab-Rashid, Tabriz, Iran; 3 Neurosciences Research Center (NSRC), Tabriz University of Medical Sciences, Tabriz, Iran

**Keywords:** Anxiety, Chronic mild stress, Cortisol, Depression, Troxerutin

## Abstract

**Objective(s):**

Chronic stress has been linked to the pathophysiology of mood disorders including anxiety and depression. In this study, we aimed to investigate the effect of troxerutin (TRX), as a flavonol, on stress-induced anxiety and depression.

**Materials and Methods:**

56 animals were randomly divided into seven groups (n=8 per group) as follows: control, saline, TRX 50, TRX 150, TRX 300, Diazepam, and Imipramine. Chronic mild stress (CMS) was induced by restraining animals in Plexiglas cylinders for 1 hr each day for 25 consecutive days. Different doses (50, 150, and 300 mg/kg, oral gavage) of troxerutin was gavaged for 14 consecutive days. At the end of treatments, anxiety- and depressive-like behaviors were tested using elevated plus-maze (EPM), open field test (OFT), and forced swimming test (FST).

**Results:**

CMS significantly increased immobility (*P*<0.05) and decreased swimming (*P*<0.01) time in FST. However, different doses of troxerutin significantly decreased immobility (*P*<0.01) and increased swimming (*P*<0.001) time. CMS also significantly (*P*<0.01) decreased the percentage of open arm entrance (%OAE), whereas troxerutin significantly increased both %OAE and percentage of open arm time (%OAT) in the EPM. Moreover, CMS significantly decreased time spent in the center (*P*<0.001) and the number of center entrances (*P*<0.01) in the OFT. However, troxerutin significantly increased time spent in the center and number of the entrances crossing. Furthermore, CMS significantly increased serum cortisol levels and troxerutin decreased it.

**Conclusion:**

Troxerutin demonstrated anxiolytic- and antidepressant-like activities in rodents, which supports the use of herbal medicine in the mood disorders.

## Introduction

Chronic stress is known to be one of the most significant environmental factors in the etiology of mood disorders including anxiety and depression. Stress is described as physiological changes that arise from response to new or threatening stimuli, which are crucial for survival. Extreme or sustained exposure to stress, in contrast, leads to a variety of neuropsychiatric disorders (1). Chronic stress-induced anxiety disorders have caused significant public health concerns recently. Anxiety is among the most common mental disorders, which is highly prevalent globally (2). 

Several chronic stress models including forced swimming, noise stimulus, electric foot shock stress, and movements restraining have been developed to induce anxiety and depression (3, 4). Restraint stress is one of the commonly used experimental stress models. This model induces physical and psychological stress at the same time by placing the animal in a plastic tube to preventing its movements (5, 6). Several studies have also reported that chronic restraint stress induces anxiety- and depressive-like behaviors in rodents (6, 7). 

The neuroendocrine response to acute and chronic stresses is mainly mediated by the hypothalamus pituitary adrenal (HPA) axis (8). This axis plays an important role in the pathophysiology of stress-related diseases such as depression and anxiety (9). Several lines of animal and human studies demonstrated that chronic stress is associated with hyperactivity of HPA axis and high plasma glucocorticoid levels (10, 11).

Troxerutin, vitamin P4; 3’,4’,7’-Tris[O-(2-hydroxyethyl)] rutin, is a natural bioflavonoids rutin, which can be isolated from the Japanese pagoda tree (12, 13). Troxerutin is also found in tea, coffee, cereals, fruits and vegetables (14, 15). Troxerutin can be easily absorbed by the gastrointestinal system and has a variety of biological activities including anti-oxidative, anti-inflammatory, and anti-thrombolytic properties (16-18). Previous experiments confirmed tissue protective effect of troxerutin in the kidney (14), liver (19), and brain (20, 21) injuries. Troxerutin has also been shown to reverse CNS insulin resistance and reduces reactive oxygen species induced by a high-cholesterol diet (20). Previous studies also showed that flavonols can alleviate anxiety and fear response induced by posttrumatic stress disorders (PTSD) in animal models (22). Moreover, antidepressant effect of flavonols in the chronic stress model has been reported (23-25).

To our knowledge, the impact of troxerutin treatment on anxiety and depressive-like behaviors has not yet been investigated in stress-exposed rats. Consequently, the aim of the present study was to investigate the effect of chronic troxerutin treatment on chronic stress-induced depression - and anxiety - like behaviors.

## Materials and Methods

***Animals***

Fifty six adult male Wistar rats, weighing 200 g to 250 g, were obtained from animal house of Tabriz University of Medical Sciences. Animals were maintained at a temperature of 23–25 on a 12 hr light-dark cycle (light on from 07:00 a.m. to7:00 p.m.) and were fed *ad libitum* with commercially available rat chow and water. Rats were given one week to acclimate to the laboratory conditions. All procedures were conducted in accordance with guidelines of care and use of laboratory animals of Tabriz University of Medical Sciences approved by Ethics Committee of Animal Research of Tabriz University of Medical Sciences.

***Drugs and treatments***

Animals were randomly divided into 7 groups (n=8 per group) as follows: control (intact), saline, TRX 50, TRX 150, TRX 300, Diazepam, and Imipramine.

**Figure 1:**
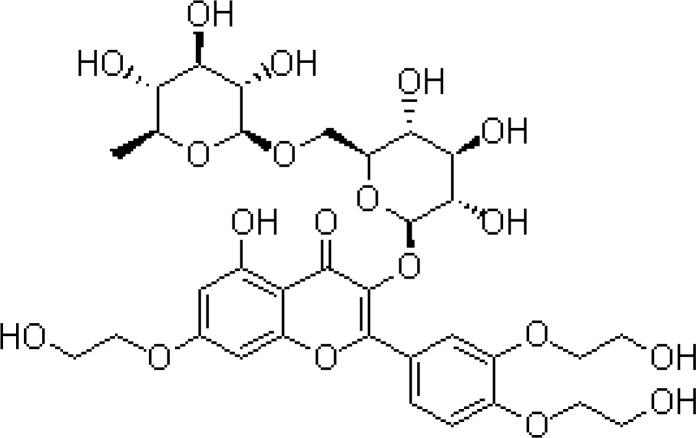
Chemical structure of Troxerutin

Animals in the saline group received chronic restraint stress for 25 days and treated with saline. Diazepam (Sigma, Germany) (1 mg/kg), as a positive control for its anxiolytic effect, was injected intraperitoneally 30 min before the tests. Imipramine (Novartis Pharmaceutical Industry, Switzerland) was administered as a positive control for its antidepressant effect intraperitoneally at 20 mg/kg once a day for 14 days (26). All drugs were administered in a final volume of 1 ml/kg. Troxerutin (Merck, Germany), (50, 150, and 300 mg/kg) was gavaged for 14 consecutive days. Chemical structure of troxerutin is shown in Figure 1.

***Chronic mild stress***

**Figure 2:**
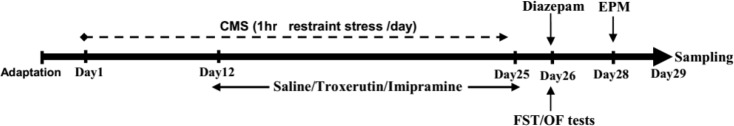
Timeline diagram of the experiments. Animals exposed to chronic mild stress (CMS) procedure for 25 days. Troxerutin treatment (50, 150 and 300 mg/kg) was started at day 12 after the CMS induction. Other groups received saline or imipramine once a day for 14 days, or diazepam treatments 30 min before the behavioral test. The following days of the last treatment, rats were subjected to forced swimming test (FST), open field test (OFT), and elevated plus-maze (EPM). Blood sampling was performed on the last day of experiments to measure serum cortisol levels

Chronic mild stress (CMS) was induced by restraining in a well-ventilated Plexiglas cylinder (25 cm length, 7 cm diameter, 0.5 cm wall) for 1 hr each day (2.00 p.m. to3.00 p.m. each day) for 25 consecutive days. All stress sessions were carried out in a nearby room to the animals’ facility, and animals were not physically compressed and did not experience pain. A summary of the experimental time points is provided in Figure 2.

***Forced swimming test***

A forced swimming test (FST) is usually used for the assessment of depressive-like behaviors in rodents. On the last day of chronic stress induction, rats were submitted to FST to evaluate depression-like behaviors. Animals were transferred to the behavioral test room 30 min prior to the test. All behavioral tests were performed between 2:00 p.m. and 6:00 p.m. under low illumination. Briefly, rats were placed individually in a transparent Plexiglas cylinder (height 50 cm, diameter 25 cm) filled with water (25 ºC) to a height of 30 cm. One day following the first trial of 15 min, a second trial was performed, which lasted 6 min. Following each swim trial, rats were removed from the cylinder and dried with towels then returned to their home cages. Water was changed between each swim session. All session were videotaped and a blinded unbiased experimenter analyzed video recording. Three different parameters were scored including immobility time (doing only those movements necessary to keep the head above the water and floating without struggling), swimming time (active swimming motions and moving around in the cylinder or diving), and climbing time (intense movements with its forepaws in and out of the water, usually directed against the walls) (27). 

***Elevated plus-maze***

The elevated plus-maze (EPM) test is one of the frequently used methods for investigation of anxiety-like behaviors in rodents. The apparatus was made from dark-painted wood and consisted of two open arms (50×10 cm), two closed arms (50×10×20 cm) and a central platform (10×10 cm), which elevated to a height of 50 cm. The maze was placed in the center of a quiet room and testing was performed under indirect dim light. Animals were individually placed on the central platform facing an open arm and allowed to explore the maze for 5 min then removed. An entry into an open arm was defined as all four paws crossing the center of the maze. The apparatus was cleaned with ethanol 70% between each test. All session were recorded by video camera, and three parameters including the percentage of open arm entries (%OAE), the percentage of time spent in open arms (%OAT), and the number of total arm entries (TAE) into the four arms were determined (28).

The formula for % OAT and %OAE calculation were as follows: a) %OAT: time spent in the open arms/total time spent in any arms × 100; b) %OAE: number of entries in open arms/total entries into any arms ×100. The number of TAE was considered as the index of locomotors activity (28).

**Figure 3:**
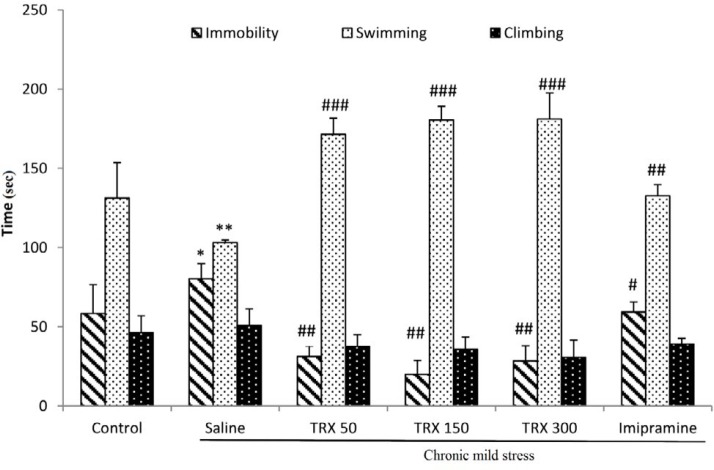
The effects of different doses (50, 150, and 300 mg/kg) of troxerutin on stress-induced depressive-like behaviors in the forced swimming test. Data are represented as means ± SEM (n=8). One-way ANOVA followed by Tukey’s post hoc test, * *P*<0.05, ** *P*<0.01 vs. control, # *P*<0.05, ## *P*<0.01, ### *P*<0.001 vs. saline group. TRX: troxerutin

**Figure 4:**
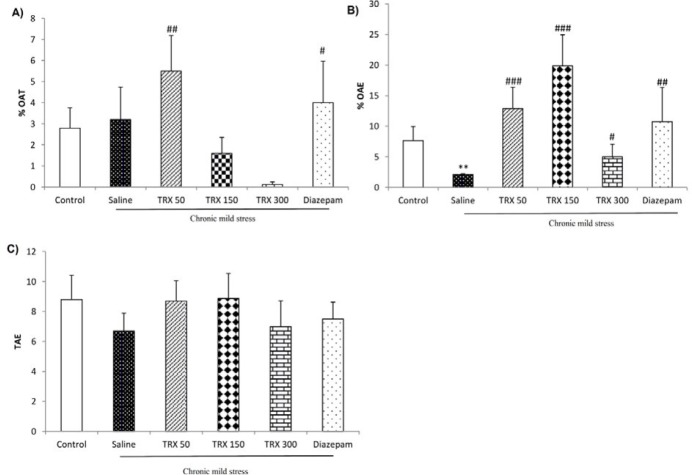
The effects of different doses (50, 150, and 300 mg/kg) of troxerutin on (A) percentage of open arm time (%OAT), (B) percentage of open arm entries (%OAE), and (C) total arm entries (TAE) in the elevated plus-maze test. Data are represented as means ± SEM (n=8). One-way ANOVA followed by Tukey’s post hoc test, ** *P*<0.01 vs. control, # *P*<0.05, ##* P*<0.1, ### *P*<0.001 vs. saline group. TRX: troxerutin

***Open field test***

The open field test (OFT) is often used to evaluate anxiety-like behaviors in rodents. The open field consisted of a four-sided arena (50×50×40 cm) with black colored wooden wall and a white floor divided into 25 squares (5×5 cm) by black lines. Each rat was placed individually in the center of the arena and left to freely explore for 5 min. Between each session, the apparatus was cleaned with 70% ethanol and dried. All sessions were video recorded and a blinded unbiased experimenter analyzed video recording. The animals’ locomotor activity was indicated by the number of crossed squares with all paws crossing, time spent in the central squares (sec), and the number of entrance to the center square (29).

***Measurement of serum cortisol***

One day following the behavioral tests, animals were deeply anesthetized with an injection of ketamine (75 mg/kg) and xylazine (10 mg/kg); then blood samples were obtained from the heart apex immediately. To avoid fluctuations in cortisol concentrations due to circadian rhythms, blood samples were collected between 9:00 a.m. to 10:00 a.m. Blood samples were centrifuged at 4000 r.p.m. for 10 min , and the separated serum samples stored at -70°C. Serum cortisol level was assayed by enzyme-linked immunosorbent assay (ELISA) kit (IBL International, Germany) according to the manufacturer’s instructions. 

***Statistical analysis***

All data are expressed as means ± SEM. For statistical comparison of the data SPSS statistics 16.0 software was used. The statistical analysis of data obtained from behavioral tests, and serum cortisol levels between groups were carried out by one-way ANOVA followed by Tukey’s *post hoc* test. For all cases, the significance level was set at *P*<0.05. 

## Results

***Effect of different doses of troxerutin on stress-induced depressive-like behaviors in the FST***

As it is shown in Figure 3, CMS induction significantly increased immobility time (*P*<0.05) and reduced swimming time (*P*<0.01) in comparison with the control group. All three different doses of troxerutin significantly decreased the immobility time (*P*<0.01) and increased the duration of swimming (*P*<0.001) in the FST compared to the saline group. Moreover, imipramine-treated animals showed lower immobility (*P*<0.05) and higher swimming time (*P*<0.01) than saline group. In addition, there were no significant differences among the groups in climbing behavior. 

***Effect of different doses of troxerutin on stress-induced anxiety-like behaviors in the EPM***

CMS induction significantly (*P*<0.01) decreased %OAE in the EPM test; however, there was no significant difference in the %OAT between saline and control groups (Figure 4 A and B). Nevertheless, troxerutin at a dose of 50 mg/kg significantly increased both %OAT (*P*<0.01) and %OAE (*P*<0.001). Moreover, troxerutin significantly increased % OAT at doses of 150 mg/kg (*P*<0.001) and 300 mg/kg (*P*<0.05) as compared to the saline group. Diazepam also significantly increased %OAT (*P*<0.05) and %OAE (*P*<0.01) as compared to the saline group (Figure 4 A and B). TAE was not affected by stress induction or treatments, indicating that there was no difference in locomotor activity among the groups (Figure 4C).

***Effect of different doses of troxerutin on stress-induced anxiety-like behaviors in the OFT***

**Figure 5:**
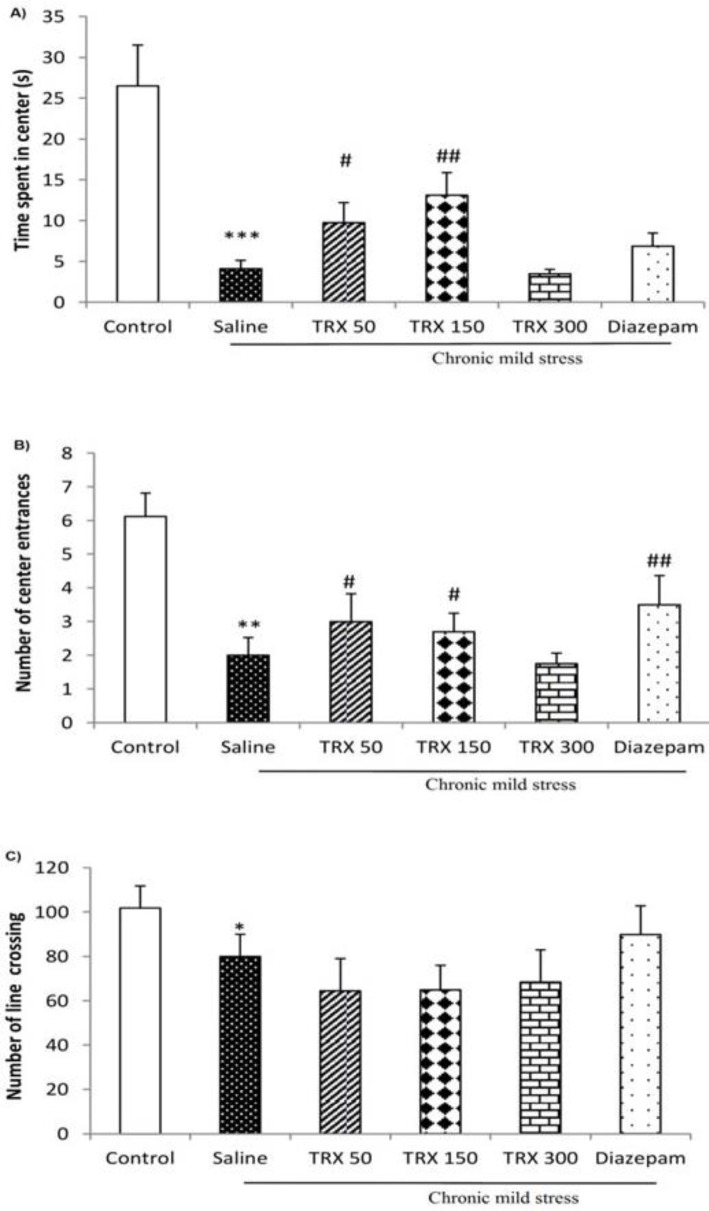
The effects of different doses (50, 150, and 300 mg/kg) of troxerutin on the anxiety-like behaviors in the open field test: (a) time spent in the center, (b) the number of entrances into the center, and (c) line entrance number. Data are expressed as means ± SEM (n=8); *P<0.05, **P<0.01, *** *P*<0.001 vs. control, # *P*<0.05, ## *P*<0.01 vs. saline group. TRX: troxerutin

The results of the OFT revealed that CMS significantly decreased time spent in the center (*P*<0.001, Figure 5A), the number of center entrances (*P*<0.01, Figure 5B), and number of line crossing (*P*<0.05, Figure 5C) as compared to the control group. Nevertheless, troxerutin at the dose of 50 mg/kg significantly (*P*<0.05) increased both time spent in the center and the number of center entrances as compared to the saline group. Also, troxerutin at the dose of 150 mg/kg significantly increased time spent in the center (*P*<0.01) and the number of center entrances (*P*<0.05) as compared to the saline group. 

Moreover, diazepam, as a positive control, significantly increased the number of center crossing (*P*<0.01) as compared to the saline group.

***Effect of different doses of troxerutin on stress-induced serum cortisol changes***

**Figure 6:**
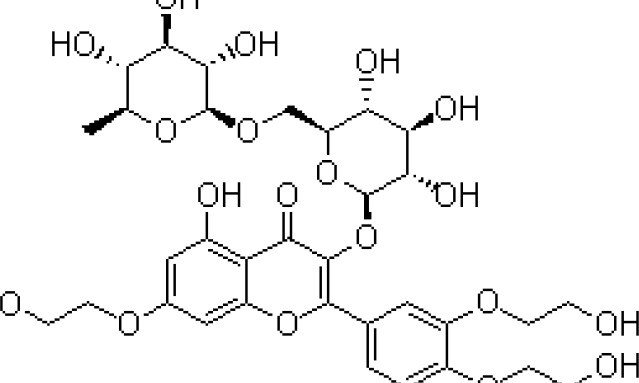
The effects of chronic mild stress (CMS) and different (50, 150, and 300 mg/kg) doses of troxerutin on serum cortisol levels. Data are expressed as means ± SEM (n=8). One-way ANOVA followed by Tukey’s *post hoc *test, *** *P*<0.001 vs. control, # *P*<0.05, ## *P*<0.01, ### *P*<0.001 vs. saline group. TRX: troxerutin

One-way ANOVA test revealed significant differences among the groups in serum cortisol levels (Figure 6). 

*Post hoc* analysis showed that 25 days of restraint procedures caused a significant (*P*<0.001) increase in cortisol levels in the stress group as compared to the control. However, chronic troxerutin administration reduced cortisol levels significantly in a dose-dependent manner. Serum cortisol levels were significantly loweredin animals after chronic administration of troxerutin 50 mg (*P*<0.001), 150 mg/kg (*P*<0.01), and 300 mg/kg (*P*<0.05) when compared to the saline group. Moreover, serum cortisol levels were significantly (*P*<0.01) reduced both in the imipramine and diazepam groups compared to saline group. 

## Discussion

In the current study, exposure to chronic restraint stress led to anxiety-like behaviors, confirmed by the fact that stress-exposed rats demonstrated a decreased number of entries into the open arms and the time spent in the open arms in the EPM. OFT findings confirmed the results of EPM test possibly due to anxiogenic effects of CMS. The results of OFT demonstrated that stressed group showed significantly reduced preference for the center of the open field as measured by reduced number of entries into the center and the time spent in the center. However, chronic administration of troxerutin significantly reduced anxiety-like behaviors both in the EPM and OFT. Thus, these results suggest that troxerutin probably has an anxiolytic activity. 

Elevated plus-maze is widely used for assessing anxiety-like behaviors in laboratory animals, which is based on the natural aversion of rodents of the elevation and open spaces (30). According to previous studies, repeated stress increases anxiety-like behaviors in rodents (5, 6, 31). Many studies have also reported that stress leads to decrease in the time spent and number of entries into the open arms of the EPM as compared with non-stressed rats (32). Similar study showed that both unpredictable CMS and chronic restraint stress for 4 weeks can lead to anxiety-like behaviors on OFT in mice (33). 

The FST is also a reliable method for investigation of antidepressant-like activity and provides information about mood in rodents (34). In this test, immobility behaviors are comparable to a state of depressed mood or helplessness in depressed humans (35). Consistent with previous findings (6, 36), the current study showed that chronic stress increased immobility time in the FST, whereas troxerutin treatment significantly decreased immobility and increased swimming time in the FST, suggesting an antidepressant-like activity. 

Troxerutin is a natural flavonoid rutin, which has well-known anti-oxidant and anti-inflammatory properties (14, 18). Several lines of evidence demonstrated that inflammatory processes are involved in the pathophysiology of chronic stress-induced depression (37). Inflammation also has a central role in mediating many of the behavioral and neuroendocrine effects of stress (37). Previous studies supported the effectiveness of anti-inflammatory agents in the treatment of depression (38). 

Troxerutin also protects different cell types (epithelial cells, fibroblasts, and lymphocytes) against apoptosis, necrosis, and mitotic death (39). Protective effects of troxerutin have been well-demonstrated experimentally against UVB-induced DNA damage (40). Therapeutic potentials of troxerutin have been well-demonstrated experimentally in neurological problems such as Alzheimer’s disease (41, 42) and cognitive deficits (18, 20). Therefore, it seems that troxerutin attenuated anxiety- and depression-like behaviors in stressed animals partially through its anti-oxidant, anti-inflammatory, and antiapoptotic properties. 

A series of behavioral, immunological, and neurochemical alterations arise in response to stressors (10, 43, 44). Stress generally modulates autonomic pathways and endocrine responses (45). Activation of the HPA axis is a well-known neuroendocrine response to stress (46) and circulating cortisol is a biomarker of chronic stress (47). Herein, we showed that chronic stress resulted in an elevation of serum cortisol levels in rats, which is generally in accordance with previous studies (10, 44, 48). Moreover, this hormonal change was accompanied by anxiety- and depressive-like behaviors. Alteration of the HPA functioning is one possible mechanism linking stress to anxiety and depression. It has been shown that high HPA axis activity and serum cortisol level is accompanied with depression (9, 49). Several studies have also revealed that depression is associated with higher baseline cortisol levels, blunted stress reactivity, and impaired recovery from psychological stress (50, 51).  Moreover, marked activation of the HPA axis has been reported in the depressed patients that can be normalized by antidepressant therapy (52, 53). It has also been shown that administration of glucocorticoids to adolescent mice was associated with increased anxiety-like behaviors (54). In this study, we found that troxerutin significantly declined serum cortisol levels. Therefore, it seems that troxerutin possibly shows its anxiolytic and antidepressant effect through modulation of HPA axis and decreasing of circulatory cortisol levels.

## Conclusion

In summary, our data demonstrated that troxerutin has an anxiolytic and antidepressant properties and plays an important role in the modulation of HPA axis activity. However, more researches are needed to elucidate the exact mechanisms underlying its anxiolytic-and- antidepressant-like effects in chronic stress model of rats.
